# One Step at a Time: Improving Gait Speed Measurement in a Geriatric Medicine Clinic

**DOI:** 10.3390/geriatrics8040081

**Published:** 2023-08-11

**Authors:** Kirstyn James, Matthew E. Growdon, Ariela R. Orkaby, Andrea Wershof Schwartz

**Affiliations:** 1 Department of Geriatric Medicine, Cork University Hospital, T12 DC4A Cork, Ireland; 2 New England Geriatric Research, Education and Clinical Centers, Division of Geriatrics & Palliative Care, Veteran Affairs Boston Healthcare System, Boston, MA 02130, USA; 3 Division of Gerontology, Beth Israel Deaconess Medical Center, Boston, MA 02215, USA; 4 Harvard Medical School, Boston, MA 02115, USA; 5 Division of Geriatrics, University of California San Francisco School of Medicine, San Francisco, CA 94143, USA; 6 Division of Aging, Brigham and Women’s Hospital, Boston, MA 02115, USA

**Keywords:** falls, age-friendly health system, quality improvement

## Abstract

(1) Background: Mobility assessment is a key component of the assessment of an older adult as a part of the Age-Friendly Health System (AFHS) “geriatric 4Ms” framework. Several validated tools for assessing mobility and estimating fall risk in older adults are available. However, they are often under-utilized in daily practice even in specialty geriatric medicine care settings. We aimed to increase formal mobility assessment with brief gait speed measurement in a geriatric medicine outpatient clinic using phased change interventions. (2) Methods: This quality improvement (QI) initiative was conducted in a single outpatient geriatric medicine clinic. All clinic attendees who could complete a gait speed measurement were eligible for inclusion. The outcome measure was the completion of a 4 m gait speed. Several change interventions were implemented on a phased basis using the Model for Improvement methodology during the period from December 2018 to March 2020. Statistical process control charts were used to record gait speed measurements and detect non-random shifts. (3) Results: During this QI initiative, 80 patients were seen, accounting for 142 clinic visits. In response to change interventions, gait speed measurement at clinic visits increased from a median of 25% of visits to 67% by March 2020. (4) Conclusions: Adopting an AFHS care model is an urgent and challenging task to improve the quality of care for older adults. This initiative details how to effectively incorporate a brief, validated assessment of mobility using gait speed measurement into every geriatric medicine outpatient visit and progresses implementation of the AFHS “geriatric 4Ms”. Mobility assessment can aid in identifying older adults at increased fall risk.

## 1. Introduction

### 1.1. Problem Description

Falls and changes in mobility are an indicator of potentially emerging vulnerability in an older adult. Falls are associated with serious injuries, emergency department presentations, and death [1,2]. 

Addressing changes in mobility and fall risk is an important part of every geriatric medicine clinic visit. The assessment of frail, older adults who often have multiple chronic conditions requires ample time and an inter-professional approach [3]. Time constraints in a busy outpatient clinic setting can make it challenging to complete all components of the assessment [4]. The Institute for Healthcare Improvement AFHS initiative promotes high-quality care for older adults using a structured framework known as the “4Ms” of geriatric medicine. The “4Ms” stand for matters most, mobility, mind, and medications [5]. Gait or walking speed is a rapidly administered test of mobility and assesses the “4M” domain of “mobility”. As a single measure that integrates all body systems such as sensory, musculoskeletal, and cognition, it offers insight into a patient’s overall functional status, mobility, and risk of falls [6,7,8,9]. However, as described above, gait speed is not consistently measured in outpatient clinic settings.

### 1.2. Available Knowledge

There are several validated methods for assessing mobility in an older adult [10]. Gait or walking speed is a recognized indicator of declining mobility, fall risk, and decreased survival [11]. Gait speed over a short distance is a rapidly administered test that offers an insight into not just a patient’s mobility but also the risk of frailty and poor outcomes. A 2011 pooled analysis of 9 cohort studies found that faster gait speed is associated with increased survival (pooled hazard ratio per 0.1 meters (m) per second (s), 0.88; 95% confidence interval (CI): 0.87–0.90; *p* < 0.001) [6,7,11]. A gait speed of fewer than 0.8 m/s in older adults is considered slow and is associated with poorer survival and increased frailty [11]. Slow gait speed is also associated with lower quality of life scores and social engagement [12].

There are few reported QI initiatives that focus on the implementation of gait speed in a geriatric medicine clinic. A 2019 QI project that included older United States (US) military veterans found an increase in gait speed following a structured walking program which lasted 6 weeks but did not address methods to increase gait speed measurement at clinic visits [13]. A 2021 QI initiative that also included older US veterans successfully incorporated routine gait speed measurement as a marker of frailty in a cardiology clinic [14].

### 1.3. Rationale

Despite knowledge of the utility of gait speed measurement as an indicator of falls and mortality risk, it was not measured regularly in our outpatient geriatric medicine clinic. There were many potential benefits to increasing gait speed measurement, including appropriate referral to specific therapy and fall prevention services (which are individualized based on risk of falls) and response of patients to preventive interventions prescribed in the clinic. 

This QI initiative aimed to improve gait speed measurement in our geriatric medicine clinic.

### 1.4. Specific Aims

We aimed to increase gait speed measurement at clinic visits at a single-site geriatric medicine clinic from 25% to 100% of visits in 1 year.

## 2. Materials and Methods

### 2.1. Context

The geriatric medicine clinic at Veterans Affairs (VA) Boston Healthcare System (BHS) is an inter-professional outpatient consultation clinic that provides comprehensive geriatric assessment for older US military veterans. There are 3 clinic sites that together offer Geriatrics assessments 5 days per week across VA BHS. One of these clinic sites was selected as the pilot site for this QI initiative. The inter-professional team in this clinic includes physicians, pharmacists, and social workers. The clinic is led by a board-certified attending in geriatric medicine and occurs once weekly. There are also two fellows in geriatric medicine who attend the clinic. Rotating learners include residents in general internal medicine and pharmacy and interns in social work. Clinic visits are 90 min in duration for new patient assessments and 45 min in duration for return visits. Return visits are usually scheduled 3 to 6 months apart. All attendees at the clinic who could complete a gait speed measurement were eligible for inclusion.

**Figure 1:**
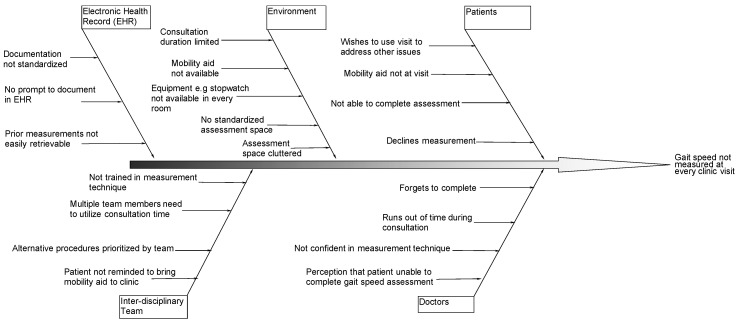
Fishbone diagram illustrating root cause analysis of reasons for not measuring gait speed at clinic visits in an ambulatory geriatric medicine clinic.

Prior to the initiation of the project, we conducted a root cause analysis of the potential barriers to consistently measuring gait speed in the clinic. A fishbone diagram method was used to explore and illustrate these barriers (Figure 1). Some of the root causes identified were subsequently selected as change interventions.

### 2.2. 4 m Gait Speed Measurement

Usual gait speed was chosen as it is a validated single measure of mobility in older adults [15]. It can be measured by any member of the inter-professional team who has received training in the measurement technique. A stopwatch or device fitted with a stopwatch (e.g., smartphone) was used to measure gait speed to the nearest 0.01 s over a straight 8 m distance free from obstacles. This consisted of a 2 m acceleration zone, 4 m assessment zone, and 2 m deceleration zone [14]. The patient was asked to walk at their normal pace and to use their assistive device and corrective eyeglasses if available. The team member measuring gait speed stood behind the patient and avoided talking during the assessment to minimize distractions. 

### 2.3. Interventions

#### 2.3.1. Study of the Interventions

Change interventions were implemented on a phased basis between December 2018 and March 2020. The change interventions were developed in conjunction with the clinic’s inter-professional team and were spaced 3 months apart. The project leadership team met regularly to review the progress of the project and the effectiveness of our interventions.

#### 2.3.2. Measures

The primary outcome measure was the percentage of visits during which gait speed was measured.

We assessed the baseline gait speed measurement in the same clinic during the period 1 April 2018 to 30 November 2018. This time period was chosen as it included baseline data when the project champions were not working in the clinic (April–June 2018) and a time period when no change interventions had been discussed with the clinic team (July–November 2018).

We reviewed electronic medical record entries for each clinic visit during the intervention period. 

#### 2.3.3. Analysis

Demographic and baseline disease characteristic data were summarized for the study population by presenting frequency distributions and descriptive statistics. Means and standard deviations (SD) were calculated for normally distributed data. Median and interquartile range (IQR) were calculated for non-normally distributed data.

Statistical process control (SPC) charts were used to monitor non-random changes from baseline median gait speed measurement and in response to the change interventions used [16]. Probability-based rules were applied to detect non-random changes in response to change interventions. SPC charts were constructed using Excel V.2018.10 (Microsoft Corporation, Redmond, WA, USA).

Study findings were reported in keeping with Standards for Quality Improvement Reporting Excellence (SQUIRE) guidelines V.2.0 [17].

#### 2.3.4. Ethical Considerations

This study was approved by the VA BHS Institutional Review Board as a quality improvement initiative under a non-research determination.

## 3. Results

Between December 2018 and March 2020, 80 patients were seen in the Geriatric Medicine clinic site accounting for 142 clinic visits. Of these, 46% (n = 37) were new referrals. The mean age was 82 ± 7 years and 98% were male. A caregiver attended the visit with 53% (n = 42) of participants. The ability to complete activities of daily living (ADLs) was recorded for all, with 51% reporting full independence for ADLs and 14% independence for all instrumental ADLs. A fall in the past 6 months was reported by 32% of patients with the number of falls ranging from 1 to 5. The median gait speed in this patient population was 0.69 m/sec (IQR: 0.56–0.87). Baseline characteristics are detailed in Table 1. 

Prior to this QI initiative, gait speed was measured at 25% of eligible assessments/clinic visits over an 8-month period. Improvements in the number of gait speed measurements performed only occurred after change interventions were implemented.

### Change Interventions

We implemented five change interventions on a phased basis. These were identified based on the root cause analysis results (Figure 1). The change in outcome measure (gait speed measurement) in response to these interventions was measured.

*Intervention 1—Stakeholder engagement and identification of project champions*

*Root cause analysis category: Inter-disciplinary team and physicians*

We identified three champions for gait speed measurement within our clinic. One was a board-certified physician in geriatric medicine who is a member of the senior leadership team in the division of geriatrics and palliative care. Two champions were geriatric medicine fellows who identified improving the quality of care for patients attending the clinic as one of their desired learning objectives. One of the champions was present at every clinic throughout the project. 

Regular and wide stakeholder engagement was conducted throughout the initiative. We met with all members of the clinic’s interprofessional team to present the findings of our fishbone diagram analysis and to discuss potential buy-in from team members for this initiative. The progress of the initiative was reviewed at weekly intervals at team huddles and clinic operational meetings.

In each clinic session, all members of the inter-professional team were reminded to measure gait speed on all patients at the pre-clinic huddle.

*Intervention 2—Adaptation of environment to optimize gait speed measurement*

*Root cause analysis category: Environment*

The clinic environment was adapted to include a designated space for gait speed measurement. The corridor in which patients walk to enter and exit the clinic visit was chosen. This corridor was straight, free from obstacles, and the appropriate length as described above. The assessment zone was demarcated to facilitate measurement. Stopwatch devices were placed in every clinic room.

*Intervention 3—Clinic electronic health record note template*

*Root cause analysis category: Electronic health record*

Permission was obtained from the division of geriatrics & palliative Care to modify the electronic health record (EHR) note template for the clinic. The physical examination portion of the template was amended to include a prompt to measure gait speed. If gait speed was not measured, an additional section was included to prompt the note writer to document why this was the case, for example, due to lack of time or the patient not having their assistive device available.

*Intervention 4—Empowering other team members to measure gait speed*

*Root cause analysis category: Inter-disciplinary team, physicians and patients*

We educated all team members on the correct technique for gait speed measurement using in-person demonstrations [10]. This broadened the number of team members available and empowered team members to remind clinicians to measure gait speed during the clinic visit. We also encouraged team members to measure gait speed at any point during the visit including the transition to and from the clinic room. 

**Figure 2:**
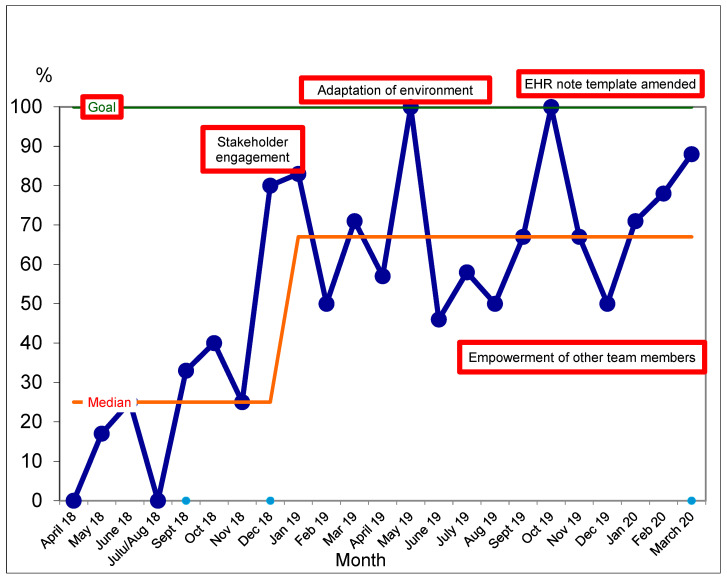
Statistical process control chart describing median gait speed measurements completed at clinic visits in an ambulatory geriatric medicine clinic. The initial median was calculated based on baseline data. Probability-based rules were applied to detect non-random changes in response to change interventions. Following the detection of a shift, a new median was calculated [18,19]. Blue dots on the x-axis denote an extension or change in the median.

These interventions resulted in an increase in gait speed measurement at clinic visits from a median of 25% of visits in December 2018 to 67% of clinic visits by March 2020 (Figure 2). 

A significant shift in median gait speed measurements was detected in May 2019 (six or more data points above the baseline median). During the period December 2019 to March 2020, we identified a potential trend toward improved gait speed measurement with 4 consecutive data points in an increasing direction. A trend is considered significant if there are five consecutive data points moving in the same direction [18]. Unfortunately, there were no in-person clinic visits in April 2020 due to the coronavirus pandemic and it could not be confirmed that this trend was significant. We explored reasons why gait speed was not measured. In 21 cases, gait speed measurement was never measured; the reported reasons for this included the patient’s assistive device being unavailable and time constraints during the consultation (Table 2). 

## 4. Discussion

### 4.1. Summary

Mobility assessment is an important component of a geriatric medicine clinic visit, and there are many validated mobility assessments [20]. Measurement of usual gait speed is a rapid and valuable assessment of mobility in the ambulatory setting and detection of slow gait speed can indicate declining mobility, risk of falls, and decreased survival [7,11]. However, there are many domains and organ systems to be assessed during a consultation with an older adult. Despite awareness of the importance of mobility assessment and its inclusion as a component of the AFHS “4Ms” framework, we identified that gait speed was only measured at 25% of visits in our clinic. A 2021 QI initiative improved gait speed measurement in an outpatient clinic setting [14]. We used a similar QI methodology to improve this and found that this inter-professional QI initiative was associated with an increase from 25% to 67% in median gait speed measurements performed at clinic visits. We aim to continue this improvement by eventually measuring gait speed at every geriatric clinic visit for all patients who can walk.

### 4.2. Interpretation

We believe several factors contributed to our results. We had strong support from the leadership team in the division of geriatrics & palliative Care. The director of outpatient clinics in the division was one of the QI initiative champions. The division chief was supportive of the project throughout and granted approval for interventions such as amending the electronic health record clinic visit template.

The project also received support from senior leadership at our institution. We aligned the goals of our project with the mission and values of the hospital. The goal of the project was to improve the quality of healthcare delivered to patients and to optimize referrals to specialties such as physical therapy which can improve quality of life. These goals were in keeping with the overall mission of our institution to deliver age-friendly care to older veterans.

The structure of the clinic’s inter-professional team was a strength during this project. All team members have assigned roles but are encouraged to speak up and contribute ideas regardless of professional background. The pre-clinic huddle is a dedicated forum for this. The enthusiasm of our team members and team culture were influential in the sustainability of this project.

We have now begun to expand this project into other geriatric medicine clinics within our institution. We have secured stakeholder support from team members at the other clinics and are commencing further change interventions to spread the improvements to other clinics. The spread of this initiative has of course been hampered by the COVID-19 pandemic and limitations in face-to-face consultations [21].

Future directions for further spread would include improving gait speed measurement for older adults in clinics other than geriatric medicine such as primary care clinics.

This project is novel as a description of how to embed one of the AFHS “4Ms”, “mobility”, into a geriatric medicine clinic through the use of QI methodology [5,22]. 

Incorporating gait speed measurement or brief mobility assessments in geriatric medicine clinics is a necessary step in standardizing high-quality care for older adults and implementing an age-friendly health system [23]. 

### 4.3. Limitations

We identified several limitations during this project. We demonstrated improvements within a single clinic. This clinic is well-resourced with a diverse inter-professional team who were particularly motivated to work on this project. One of the geriatric medicine fellows completed a scholarship in QI and both clinic fellows were project champions. As this initiative spreads to other clinics, these champions will not be present. Identification of other appropriate champions will be necessary.

While we demonstrated an improvement in gait speed measurement, we were unable to demonstrate any other mobility-related outcomes at this time, such as reduced rates of falls, hospitalization, or death. As we continue to collect longer-term data related to the sustainability of this project, we hope to measure the association between consistent and repeated gait speed measurement and these additional outcome measures.

## 5. Conclusions

Gait speed measurement in older adults is important as an indicator of overall health and mobility. Identification of slow gait speed can prompt fall prevention strategies and referrals to specialties such as physical therapy. Despite knowing the importance of mobility assessment for older adults, we were not consistently completing this assessment or using a validated tool such as gait speed in our geriatric medicine clinic. This phased QI initiative utilized the strengths of our inter-professional team, existing resources, and phased change interventions to increase gait speed measurement in a single geriatric medicine clinic. These improvements were sustained. Our results also represent a step forward in developing an AFHS “geriatric 4Ms” approach to geriatric medicine clinic visits. As part of an overall strategy to become an AFHS, we are now spreading this initiative to other geriatric medicine clinics. We also aim to demonstrate that through leading by example, we can further spread formal gait speed measurement to other clinics and increase awareness of AFHS approaches to the care of older adults that rely on more than self-report or intuition. 

## Figures and Tables

**Table 1:** Baseline characteristics and assessment findings at clinic visits.

	N = 80
Mean age (years)	82 ± 7.7
Male	98%
Visit type	
New	46%
Follow up	54%
Patient accompanied by a caregiver	53%
Medical history	
Diabetes	38%
Dementia	51%
Social Circumstances	
Lives alone (n = 63)	38%
Caregiver stress (n = 64)	25%
ADLs	
Independent in all	51%
Requries assistance, some/most/all	38%
Dependent, some or most	6%
Dependent, all	5%
iADLs	
Independent, all	14%
Requires assistance, some/most/all	24%
Dependent, some or most	36%
Dependent, all	26%
Mobility	
Fall in the past 6 months	32%
Number of falls in past 6 months (range)	1–5
Median gait speed	0.69 (IQR: 0.56–0.87)
Orthostatic hypotension (n = 49)	12%
Ability to don/doff socks (n = 72)	
Able	54%
With difficulty	24%
Unable	22%
Assistive device use	58%
Assistive device type (n = 43)	
Cane	40%
Walker	42%
Wheelchair	18%
Type of shoes (n = 50)	
Laces	50%
Slip-on	28%
Velcro	22%
Chair stand (n = 69)	
Able	39%
With difficulty	28%
Unable	33%

**Table 2:** Reasons for not measuring gait speed reported in an ambulatory geriatrics clinic.

n = 21	% (n)
No assistive device	33% (7)
Wheelchair user	38% (8)
No time	10% (2)
Other/not documented	19% (4)

## Data Availability

Data is unavailable due to privacy restrictions.

## References

[1] Choi N.G., Choi B.Y., DiNitto D.M., Marti C.N., Kunik M.E. (2019). Fall-related emergency department visits and hospitalizations among community-dwelling older adults: Examination of health problems and injury characteristics. BMC Geriatr..

[2] Burns E., Kakara R. (2018). Deaths from Falls Among Persons Aged ≥65 Years—United States, 2007–2016. MMWR Morb. Mortal. Wkly. Rep..

[3] Ellis G., Whitehead M.A., Robinson D., O’Neill D., Langhorne P. (2011). Comprehensive geriatric assessment for older adults admitted to hospital: Meta-analysis of randomised controlled trials. BMJ.

[4] Morley J.E., Little M.O., Berg-Weger M. (2017). Rapid Geriatric Assessment: A Tool for Primary Care Physicians. J. Am. Med. Dir. Assoc..

[5] Fulmer T., Mate K.S., Berman A. (2018). The Age-Friendly Health System Imperative. J. Am. Geriatr. Soc..

[6] Middleton A., Fritz S.L., Lusardi M. (2015). Walking speed: The functional vital sign. J. Aging Phys. Act..

[7] Studenski S., Perera S., Wallace D., Chandler J.M., Duncan P.W., Rooney E., Fox M., Guralnik J.M. (2003). Physical performance measures in the clinical setting. J. Am. Geriatr. Soc..

[8] Zhang Y.R., Xu W., Zhang W., Wang H.F., Ou Y.N., Qu Y., Shen X.N., Chen S.D., Wu K.M., Zhao Q.H. (2022). Modifiable risk factors for incident dementia and cognitive impairment: An umbrella review of evidence. J. Affect. Disord..

[9] Lauretani F., Russo C.R., Bandinelli S., Bartali B., Cavazzini C., Di Iorio A., Corsi A.M., Rantanen T., Guralnik J.M., Ferrucci L. (2003). Age-associated changes in skeletal muscles and their effect on mobility: An operational diagnosis of sarcopenia. J. Appl. Physiol..

[10] James K., Schwartz A.W., Orkaby A.R. (2021). Mobility Assessment in Older Adults. N. Engl. J. Med..

[11] Studenski S., Perera S., Patel K., Rosano C., Faulkner K., Inzitari M., Brach J., Chandler J., Cawthon P., Connor E.B. (2011). Gait speed and survival in older adults. JAMA.

[12] Ekström H., Dahlin-Ivanoff S., Elmståhl S. (2011). Effects of walking speed and results of timed get-up-and-go tests on quality of life and social participation in elderly individuals with a history of osteoporosis-related fractures. J. Aging Health.

[13] Espinoza S.E., Orsak B., Wang C.P., MacCarthy D., Kellogg D., Powers B., Conde A., Moris M., Padala P.R., Padala K.P. (2019). An Individualized Low-Intensity Walking Clinic Leads to Improvement in Frailty Characteristics in Older Veterans. J. Frailty Aging.

[14] Orkaby A.R., James K., Leuchtenburg J., Solooki E., Gaziano J.M., Driver J.A. (2021). Taking prevention to the next step: Implementation of a brief, sustainable frailty assessment in a cardiology clinic. BMJ Open Qual..

[15] Rydwik E., Bergland A., Forsén L., Frändin K. (2012). Investigation into the reliability and validity of the measurement of elderly people’s clinical walking speed: A systematic review. Physiother. Theory Pract..

[16] Benneyan J.C., Lloyd R.C., Plsek P.E. (2003). Statistical process control as a tool for research and healthcare improvement. Qual. Saf. Health Care.

[17] Ogrinc G., Davies L., Goodman D., Batalden P., Davidoff F., Stevens D. (2016). SQUIRE 2.0 (Standards for QUality Improvement Reporting Excellence): Revised publication guidelines from a detailed consensus process. BMJ Qual. Saf..

[18] Perla R.J., Provost L.P., Murray S.K. (2011). The run chart: A simple analytical tool for learning from variation in healthcare processes. BMJ Qual. Saf..

[19] Swed F.S., Eisenhart C. (1943). Tables for Testing Randomness of Grouping in a Sequence of Alternatives. Ann. Math. Stat..

[20] Soubra R., Chkeir A., Novella J.L. (2019). A Systematic Review of Thirty-One Assessment Tests to Evaluate Mobility in Older Adults. Biomed. Res. Int..

[21] Schwartz A.W., Driver J.A., Pollara L.M., Roefaro J., Harrington M.B., Charness M.E., Skarf L.M. (2022). Increasing Telehealth Visits for Older Veterans Associated with Decreased No-Show Rate in a Geriatrics Consultation Clinic. J. Gen. Intern. Med..

[22] Tinetti M., Huang A., Molnar F. (2017). The Geriatrics 5M’s: A New Way of Communicating What We Do. J. Am. Geriatr. Soc..

[23] Burke R.E., Brown R.T., Kinosian B. (2022). Selecting implementation strategies to drive Age-Friendly Health System Adoption. J. Am. Geriatr. Soc..

